# Cause and risk factors of early neonatal death in Ethiopia

**DOI:** 10.1371/journal.pone.0275475

**Published:** 2022-09-29

**Authors:** Neamin Tesfay, Rozina Tariku, Alemu Zenebe, Zewdnesh Dejene, Fitsum Woldeyohannes

**Affiliations:** 1 Centre of Public Health Emergency Management, Ethiopian Public Health Institutes, Addis Ababa, Ethiopia; 2 Health Financing Program, Clinton Health Access Initiative, Addis Ababa, Ethiopia; University of Mississippi Medical Center, UNITED STATES

## Abstract

**Background:**

Globally, three fourth of neonatal deaths occur during the early neonatal period, this makes it a critical time to reduce the burden of neonatal death. The survival status of a newborn is determined by the individual (neonatal and maternal), and facility-level factors. Several studies were conducted in Ethiopia to assess early neonatal death; however, most of the studies had limited participants and did not well address the two main determinant factors covered in this study. In response to this gap, this study attempted to examine factors related to early neonatal death based on perinatal death surveillance data in consideration of all the possible determinants of early neonatal death.

**Methods:**

The national perinatal death surveillance data were used for this study. A total of 3814 reviewed perinatal deaths were included in the study. Bayesian multilevel parametric survival analysis was employed to identify factors affecting the survival of newborns during the early neonatal period. Adjusted time ratio (ATR) with 95% Bayesian credible intervals (CrI) was reported and log-likelihood was used for model comparison. Statistical significance was declared based on the non-inclusion of 1.0 in the 95% CrI.

**Result:**

More than half (52.4%) of early neonatal deaths occurred within the first two days of birth. Per the final model, as gestational age increases by a week the risk of dying during the early neonatal period is reduced by 6% [ATR = 0.94,95%CrI:(0.93–0.96)]. There was an increased risk of death during the early neonatal period among neonates deceased due to birth injury as compared to neonates who died due to infection [ATR = 2.05,95%CrI:(1.30–3.32)]; however, perinates who died due to complication of an intrapartum event had a lower risk of death than perinates who died due to infection [ATR = 0.87,95%CrI:(0.83–0.90)]. As the score of delay one and delay three increases by one unit, the newborn’s likelihood of surviving during the early neonatal period is reduced by 4% [ATR = 1.04,95%CrI:(1.01–1.07)] and 21% [ATR = 1.21,95%CrI:(1.15–1.27)] respectively. Neonates born from mothers living in a rural area had a higher risk of dying during the early neonatal period than their counterparts living in an urban area [ATR = 3.53,95%CrI:(3.34–3.69)]. As compared to neonates treated in a primary health facility, being treated in secondary [ATR = 1.14,95%CrI:(1.02–1.27)] and tertiary level of care [ATR = 1.15,95%CrI:(1.04–1.25)] results in a higher risk of death during the early neonatal period.

**Conclusion:**

The survival of a newborn during the early neonatal period is determined by both individual (gestational age, cause of death, and delay one) and facility (residence, type of health facility and delay three) level factors. Thus, to have a positive early neonatal outcome, a tailored intervention is needed for the three major causes of death (i.e Infection, birth injury, and complications of the intrapartum period). Furthermore, promoting maternal health, improving the health-seeking behaviour of mothers, strengthening facility readiness, and narrowing down inequalities in service provision are recommended to improve the newborn’s outcomes during the early neonatal period.

## Introduction

Neonatal mortality is one of the prominent indicators of the health and economic status of a nation [1]. Globally, neonatal mortality has dropped by 52%, from 5 million in 1900 to 2.4 million in 2019; however, the pace of decline was lower as compared to the post-neonatal period [2]. Comparatively, a very high burden of neonatal mortality was observed in sub-Saharan African and South Asian countries [3]. Several global initiatives were launched to reduce neonatal mortality, Every Newborn Action Plan (ENAP) is one of the strategies adopted to prevent newborns death based on evidence-based solutions. In addition, strategies aimed at ending preventable maternal mortality were also put in place to reduce inequality in service access and provision [4, 5]. To consolidate this effort, a new global target was established under the sustainable development goal (SDG), which has a goal of reducing neonatal mortality to only 12 deaths per 1000 live births by 2030 [6].

Ethiopia stood on the 5^th^ rank among countries with the highest number of neonatal death in the world [7]. Despite making progress in the last two decades [8], the country’s progress in reducing neonatal death was much slower compared to the post-neonatal period [9]. According to the Ethiopian Demographic and Health Survey (EDHS), there was a decline in the neonatal mortality rate, from 49 death per 1000 live births (LBs) in 2000 to 33 deaths per 1000 live births (LBs) in 2019 [10]. However, the presence of noticeable regional variation has slowed the progress in the reduction of neonatal mortality in Ethiopia [11].

One of the key strategies ENAP put in place to reduce the high neonatal mortality is producing real-time information on every newborn’s death, generating policy directions that would improve the quality of service, and averting similar deaths in the future [12]. Many underdeveloped countries lack effective civil registration and vital statistics (CRVS) systems to register and count all deaths and assign causes to the deaths [13]. This triggered the need for the establishment of a surveillance system using existing health system platforms [12]. In light of this, Ethiopia established the perinatal death surveillance and response system (PDSR) in 2017, which was integrated into the pre-existing maternal death surveillance and response system (MDSR) [14]. Accordingly, in August 2017, the MDSR system was changed to a more comprehensive Maternal and Perinatal Death Surveillance and Response (MPDSR) by adding the extended definition of perinatal death [15]. MPDSR system is a continuous-action cycle designed to provide real-time, actionable data on maternal and perinatal mortality levels, causes of death, and contributing factors, with a primary objective of using the findings to plan appropriate and effective preventive actions [16]. The preventive actions are expected to be taken at different levels, at a health facility level actions are aimed at addressing the barriers related to the health care providers. While at national and subnational levels the actions are related to designing programmatic level responses [17]. MPDSR operates at both facility and community levels; however, the system implementation has been challenged by low engagement of the community, underreporting, and poor utilization of the data to make evidence-based decisions [18].

Globally, 75% and 42% of neonatal death occur within the first seven days and the first 24 hours of birth respectively [19]. As a result, the early neonatal period has become more critical than ever before. Both globally and in the Ethiopian setup, nearly 80% of deaths are due to preterm birth, intrapartum events such as birth asphyxia, and infections [20]. Interventions focused on early neonatal death will have a significant contribution to the reduction of the overall neonatal mortality rate [19].

In general, early neonatal mortality is determined by three fundamental elements: namely Maternal factor, neonate factor, and the health facility’s level of readiness [21]. Gestational age, sex, birth weight, birth order, malpresentation (including birth injury), monochorionic pregnancy, and congenital malformations are some of the neonatal factors that could affect the survival of early neonates [22–29]. Birth interval, history of Antenatal care (ANC), early breastfeeding initiation practice, maternal age, maternal parity, health status (both during and before pregnancy), education status, residence, and wealth index are some of the maternal factors that determine the survival status of early neonates [30–34]. On the other hand, early diagnosis of labour, proper follow-up of labour, strong referral, and availability of essential supply and manpower are potential facility-level factors that affect the outcome of early neonates [35–40]. Several studies were conducted in Ethiopia on early neonatal death; however, most of the studies were focused on small geographical areas and were unable to address the three pillars of early neonatal death in their study. Thus, this study aims to identify factors that determine the death of a neonate from birth to seven days after birth by incorporating both individual and facility-level factors among nationally reviewed perinatal deaths.

## Methods and materials

### Study setting

Ethiopia has an estimated population of 117,876,000 in 2021, out of which 17, 216,372 were under-five children [41, 42]. Administratively, Ethiopia has ten regions and two city administrations, namely Tigray, Afar, Amhara, Oromia, Somali, Benishangul-Gumuz, Southern Nations Nationalities and Peoples Region (SNNPR), Sidama, Gambella, Harari, Addis Ababa city administration and Dire Dawa city administration [43]. Ethiopia has a total of 17,187 health posts,4026 public health facilities(3,724 health centers and 302 public hospitals), and 5,401 private health facilities [43]. In addition, the country has high infant, under-five, and maternal mortality (47 per 1000LBs), (59 per 1000LBs), and (412 per 100,00 LBs), respectively [10, 44].

### Data source and study participant

The study used data from Ethiopian Public Health Institutes (EPHI), which is collected and compiled from various health facilities across the country. The study utilized an updated programmatical and epidemiological review of perinatal death data obtained from all PDSR implementing regions for four consecutive years (2018–2021). The source population for this study was all perinate who died and were reviewed by the MPDSR committee during the study period. The system used facility-based abstraction format (FBAF) and verbal autopsy (VA) as data sources for the review process. After the data is extracted from the source a review committee prepares the final report of the reviewed death. The final report will then be sent to the national data hub through perinatal death reporting format (PDRF) by the focal person assigned in the reporting facility. A total of 3814 reviewed perinatal deaths were included in the study. Furthermore, the PDSR data was hierarchical i.e., perinate was nested in 161 reporting health facilities of the country. However, only 4%(158/4026) of expected public health facilities were actively reporting reviewed perinatal death to the national data hub at the time of the study.

### Case definition and inclusion and exclusion criteria

#### Case definition (extended perinatal death)

Death of a fetus born after 28 completed weeks of gestation or neonatal deaths through the first 28 completed days after birth [14].

#### Inclusion criteria

All death of a perinate from birth up to the first seven days of life were included in the study.

#### Exclusion criteria

Stillbirth perinates and neonates deceased after 7 days of delivery were not part of the study.

### Study variables

#### Outcome variables

The main outcome variable of this study is time to early neonatal mortality (death occurred between zero to seven days after birth) among reviewed perinatal death. Early neonatal death is the death of neonates between 1 and 7 completed days of birth. Therefore, the dependent variable in this study was defined as “the risk of death occurring between 1 to 7 days period”. The outcome variable was thus survival time in days for the neonates. A neonate who died within seven days of birth was deemed to have had the event and assigned the number ‘1’, while those who did not die within the stated period were assigned the number ‘0’.

#### Explanatory variables

Both individual (neonatal and maternal factor) and facility (community)-level variables were included as a predictor in the model. Sex, gestational age, place of birth, mode of delivery, and assigned cause of death were included as neonatal factors in the model. The medical cause of death was incorporated as an individual-level factor after the underlying cause of death was assigned using the International Classification of Diseases -Perinatal Mortality (ICD-PM) [45]. From the maternal factors, variables such as maternal age, maternal parity, educational status, number of ANC (antenatal care) visits, a score of delay one, and maternal health conditions were included in the model. Maternal health conditions were assigned per the guidance of ICD-PM. The score of delay one, which is a delay in deciding to seek care [46], was computed using the row sum of seven variables included under the domain; namely 1) family poverty,2) bad experience with previous health care,3) failed to recognize the danger of pregnancy,4) unaware where to go,5) had no one take care of other children,6) reliant on traditional practice and 7) lack of decision to a health facility. All of them were binary variables with ‘Yes’ and ‘No’ responses and after summation of the score and to keep the normality of the data a square root transformation was done [47]. Finally, the transformed variable was treated as continuous variables to make a simple and parsimonious model [48].

At a facility (community) level; variables such as residence, type of region, type of health facility, a score of delay two, and a score of delay three were taken into consideration. The type of region was classified into three categories (city, agrarian, and pastoralist) based on the cultural and socio-economic backgrounds of the population [49]. Furthermore, the type of facility was codified into three classes (primary, secondary, and tertiary facilities) according to their manpower, medical equipment, and service provision [50]. Moreover, the score of delay two (delayed arrival to health facility) and delay three (delayed provision of adequate care in a health facility) [46], were computed similarly to the score of delay one. The score of delay two was computed using four items: namely 1) absence of transportation, 2) expensive cost of transportation, 3) no facility within a reasonable distance and 4) poor road condition. Similarly, the score of delay three was also computed using four items; namely,1) long travel time from health facility to health facility, 2) long waiting time before treatment was received, 3) mistake during an assessment, diagnosis, and treatment and 4) shortage of equipment and supplies. Both delays (two and three) were measured using binary variables and the responses were set as ‘Yes’ and ‘No’ options. The delays were included in the final model in a similar fashion as delay one, i.e., they were treated as a continuous variable, to smoothen the model building process.

### Operational definition

#### Older age

Women 35 and above were considered older-aged women in the study [51]. The variable was categorized into two by assigning ‘< 35’ and ‘≥ 35’according to the criteria.

#### Grand multipara

A woman with ≥5 parity was considered a grand multipara [52]. The variable was categorized into two levels by using the ‘<5’ and ‘≥5’ options.

#### Preterm birth

It is defined as babies born alive before the 37^th^ week of pregnancy is completed [53]. The variable was categorized into two levels by using the ‘preterm’ and ‘term’ option. Moreover, cases were categorized by the time of death; antepartum, intrapartum stillbirth, unknown time of stillbirth, and neonatal death. The contributing maternal conditions were classified into five major categories (M1 to M4, with M5 representing no identified condition) per the guidance of ICD_PM_10 (Table 1) [54].

**Table 1:** ICD-PM categories with the specific cause of perinatal death and maternal health condition.

Time of death	Category	Description	Example
Antepartum death	A1	Congenital malformations and chromosomal abnormalities	Anencephaly, encephalocele, microcephaly, congenital hydrocephalus, spina bifida, etc.
	A2	Infection	Congenital syphilis, congenital malaria, congenital rubella syndrome, congenital TB, etc.
	A3	Antepartum hypoxia	Intrauterine hypoxia
	A4	Other specified antepartum disorder	Vasa previa, ruptured cord, twin-twin transfusion, Intraventricular (nontraumatic) haemorrhage, Rhesus and ABO isoimmunization, etc.
	A5	Disorders related to fetal growth	Small for gestational age, macrosomia, post-term, etc.
	A6	Antepartum death of unspecified cause	Intrauterine death of unspecified cause
Intrapartum death	I1	Congenital malformations and chromosomal abnormalities	Anencephaly, encephalocele, microcephaly, congenital hydrocephalus, spina bifida, etc.
	I2	Birth trauma	Intracranial laceration and haemorrhage due to birth injury, Fracture of skull due to birth injury, etc.
	I3	Acute intrapartum event	Intrauterine hypoxia
	I4	Infection	Congenital syphilis, congenital malaria, congenital rubella syndrome, congenital TB, etc.
	I5	Other specified intrapartum disorder	Vasa previa, ruptured cord, twin-twin transfusion, Intraventricular (nontraumatic) haemorrhage, Rhesus and ABO isoimmunization, etc.
	I6	Disorders related to fetal growth	Small for gestational age, extreme low birthweight, macrosomia, post-term, etc.
	I7	Intrapartum death of unspecified cause	Fetal death of unspecified cause
Neonatal death	N1	Congenital malformations, and chromosomal abnormalities	Anencephaly, encephalocele, microcephaly, congenital hydrocephalus, spina bifida, etc.
	N2	Disorders related to fetal growth	Small for gestational age, exceptionally large baby, post-term, etc.
	N3	Birth trauma	Cerebral haemorrhage due to birth injury, intraventricular haemorrhage due to birth injury, etc.
	N4	Complications of intrapartum events	Intrauterine hypoxia, birth asphyxia
	N5	Convulsions and disorders of cerebral status	Neonatal cerebral irritability, neonatal cerebral depression, neonatal coma, etc.
	N6	Infection	Tetanus neonatorum, bacterial meningitis, bacterial sepsis, congenital pneumonia, etc.
	N7	Respiratory and cardiovascular disorders	Respiratory distress syndrome, Neonatal aspiration syndromes, neonatal cardiac failure, neonatal cardiac dysrhythmia, neonatal hypertension, etc.
	N8	Other neonatal conditions	Vasa previa, ruptured cord, twin-twin transfusion, Rhesus and ABO isoimmunization, kernicterus, etc.
	N9	Low birthweight and prematurity	Extremely low birth weight, extreme immaturity
	N10	Miscellaneous	Cases where codes from several other sections of ICD-10 should be used
	N11	Neonatal death of unspecified cause	Congenital renal failure, termination of pregnancy, affecting fetus and newborn, withdrawal symptoms from drug
Maternal conditions	M1	Complications of placenta, cord and membranes	Abruptio placentae, prolapsed cord, chorioamnionitis, etc.
	M2	Maternal complications of pregnancy	Premature rupture of membranes, oligo- and polyhydramnios, ectopic pregnancy, multiple pregnancy, etc.
	M3	Other complications of labour and delivery	Breech delivery and extraction, forceps delivery, Caesarean delivery
	M4	Maternal medical and surgical conditions	hypertensive disorders, maternal injury, maternal use of tobacco, alcohol or drugs, etc.
	M5	No maternal conditions	No condition identified

### Data management and statistical analysis

The data was exported from Epi -info version 7.2 to Stata version 17 for data cleaning and further analysis. Both descriptive (count, mean, percentage, and standard deviation (SD)) and analytical analyses were carried out. The descriptive analysis tables were presented using three-way tables, by classifying the table using the timing of death whether the death was a stillbirth, early neonatal, and late neonatal death. The analytical analysis was carried out using Bayesian multilevel parametric survival analysis.

#### Model building

The model building process has gone through consecutive processes listed below.

**Step One:** both individual and facility-level variables were checked using bivariable Cox regression analysis, and variables that were significant at a 0.2 p-value were retained in the model for the next step of the analysis.

**Step Two:** the retained variables were assessed and checked for multicollinearity using variance inflation factor (VIF); then, the proportional hazard (PH) assumption was evaluated using a log cumulative hazard plot.

**Step Three**: After the plot, to get an objective measurement of the PH assumption, the model goodness of fit was checked using the Schoenfeld residuals. The test demonstrated that the PH assumption was violated for both the global test and rank test due to the significant correlation of time to early neonatal death(Table 2) [55].

**Table 2:** Schoenfeld residual test for proportionality assumption of the Cox model.

Variables	rho	chi^2^	df	Prob>chi2
Assigned cause of death	0.00	0	1	0.95
Sex of the perinate	0.03	1.55	1	0.21
Estimated gestational age	-0.23	104.85	1	<0.001
Place of birth	0.01	0.11	1	0.74
Maternal health condition	-0.03	1.76	1	0.18
Educational status	0.05	5.29	1	0.02
Maternal age	0.01	0.12	1	0.73
maternal parity	0.00	0.05	1	0.82
Number of ANC visit	-0.02	0.53	1	0.46
Score of delay 1	-0.02	0.86	1	0.35
Level of care	0.04	4.37	1	0.04
Residence	0.57	521.74	1	<0.001
Score of delay 2	0.00	0.06	1	0.81
Score of delay 3	0.06	8.57	1	<0.001
Global test		720.84	14	<0.001

< 0.001 means significant at a 5% significance level; the proportionality assumption is violated

**Step Four:** After the confirmation of the violation of the PH assumption, it was decided to use the accelerated failure time (AFT) model [55].

**Step Five:** Using the AFT model, the suitability of the multilevel survival model was checked, by using the null model (by excluding predicting covariates)

**Step Six:** After confirming the applicability of multilevel survival analysis, due to the presence of clustering effect, as confirmed by the significant value of the variance and intraclass correlation coefficient (ICC). The best-fitted variables were selected through the step-wise variable selection technique [56].

**Step Seven:** Finally, to obtain an unbiased and strong estimate we decided to use the Bayesian multilevel survival analysis approach.

**Step Eight:** Before running the Bayesian multilevel survival analysis, we attempted to set preconditions to run the analysis using the AFT model. The following were the major preconditions adopted

- To get an unbiased estimate of parameters in the survival analysis, the Monte Carlo Markov Chain (MCMC) method was utilized [57]. For the MCMC estimation, we used diffused prior distributions; normal priors with large variances (mean = 0, variance = 10^6^) for regression parameters; and inverse gamma (0.001, 0.001) for precision parameters.
  - We then run the chain for 50,000 iterations with a burn-in length of 25,000 iterations to ensure convergence of the Markov chains.
  - The convergence of Markov chain was assessed by a visual inspection of the trace- and autocorrelation plots.

**Step Nine:** After establishing the above-mentioned precondition of Bayesian multilevel survival analysis, five AFT models (Log-logistic, Lognormal, Exponential, Weibull, and Gompertz) were fitted to decide the final model.

**Step Ten:** Model comparison was carried out using the deviance information criteria (DIC), which is the Bayesian version of the frequentist Akaike Information Criterion (AIC) and Bayesian Information Criterion (BIC), and the Bayes factors (BF). A model with the smallest DIC value and BF>1 was selected as the best-fitted model (Table 3) [58].

**Table 3:** Model comparison parameter on time of early neonatal death in Ethiopia.

Type of model	DIC	LMR	log (BF)
Loglogistic^a^	8638.4	-4380.1	373.6
Lognormal	9204.5	-4913.0	_
Gamma	10188.1	-5409.1	-654.6
Exponential	10536.7	-5624.0	-752.4
Weibull	10428.8	-5530.5	-744.7

^a^ preferred model

**Step Eleven:** After the selection of the best-fitted model, three models were fitted again for the finally selected variables. Model 1, which does not include any covariate; Model 2, which includes individual-level covariates, and Model 3, which contains variables in Model 2 plus facility-level covariates.

**Step Twelve:** The final result of the model was reported using the posterior adjusted time ratio (ATR) with 95% Bayesian credible intervals (CrI) for each of the variables in the full model (Model 3). The posterior adjusted hazard ratio (AHR) was not utilized due to the violation of the PH assumption [59] The statistical significance was based on the non-inclusion of 1.0 in the 95% CrI.

### Ethical approval

We used secondary data obtained from EPHI with no personal identifier information of the participants. The EPHI Review Board and Public Health Emergency Management Unit approved the research proposal with Ref. No. EPHI 6_5/437. Since the study used secondary data other ethical measures were not required.

## Result

### Selected characteristics of reported facilities

**Fig 1:**
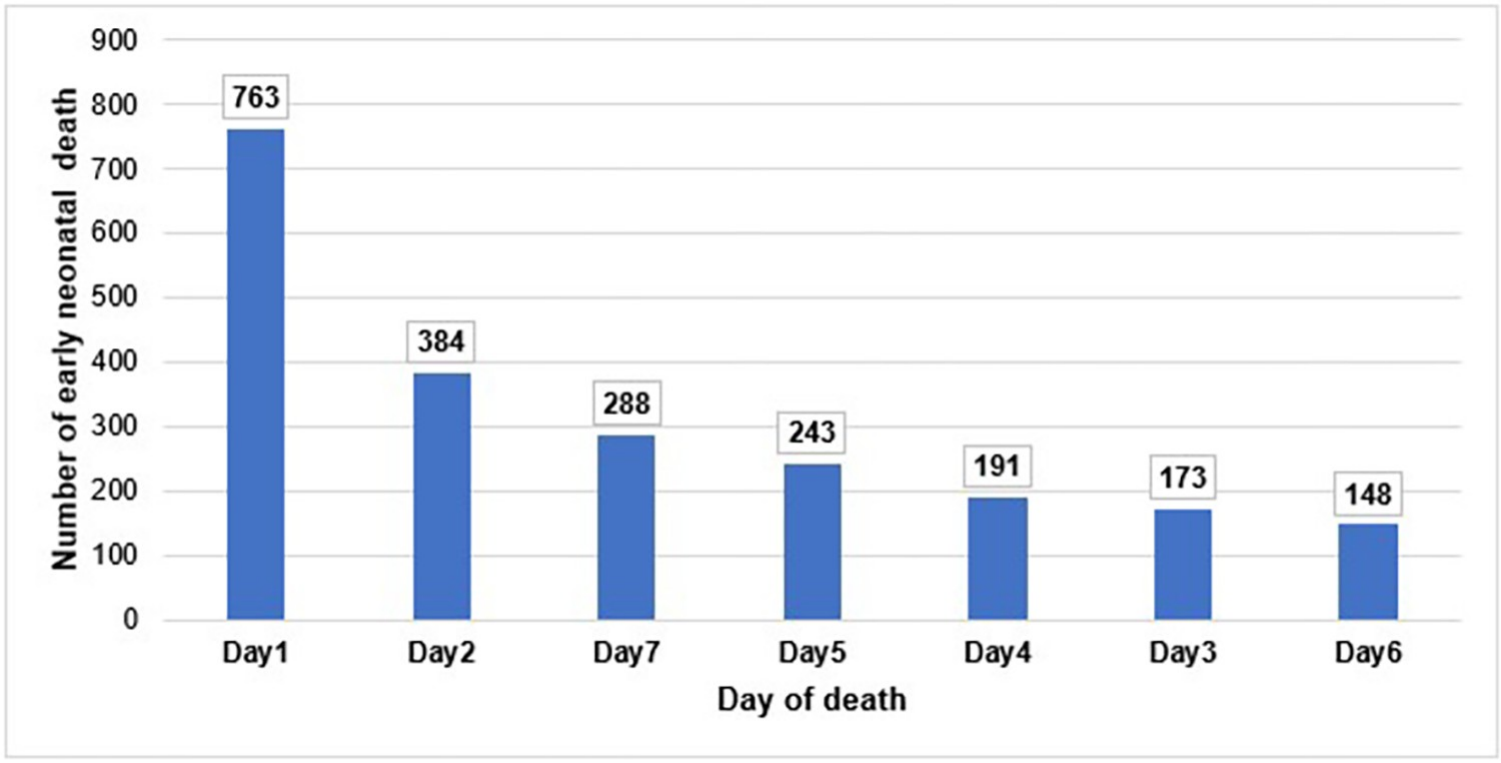
Distribution of early neonatal death by the day of stay after birth in Ethiopia,2021.

A total of 3814 perinatal deaths were included in the study, out of which 2190 (57.4%) were early neonatal deaths. Among the early neonatal deaths, 1147(52.4%) occur within the first two days after birth (Fig 1). Around 84% of the reported perinatal deaths from tertiary level health facilities were early neonatal deaths. Region-wise, 100.0%,90.5%, and 85.4% of the reported perinatal death from Gambella, Harari, and Addis Ababa respectively were early neonatal deaths. Furthermore, 69.9% of reported perinatal deaths in 2018 were early neonatal deaths (Table 4).

**Table 4:** Selected background characteristics of reporting facilities by the timing of death in Ethiopia,2021.

Characteristic	Overall, N = 3,814	Occurrence of perinatal death		
		Stillbirth (n = 1417)(%)	Early neonate (n = 2190)%	Late neonate (n = 207)(%)
Level of health care				
Primary	1,999	991(49.6)	944(47.2)	64(3.2)
Secondary	879	363(41.3)	460(52.3)	56(6.4)
Tertiary	936	639(6.7)	786(84.0)	87(9.3)
Ownership of Health facility	** **			
Public	3,801	1410(37.1)	2184(57.5)	207(5.5)
NGO	2	0 (0.0)	2 (100.0)	0 (0.0)
Private	11	7(63.6)	4(36.4)	0 (0.0)
Data source				
FBAF	3,639	1316(36.2)	2120(58.3)	203(5.6)
VA	175	101(57.7)	70(40.0)	4(2.3)
Reporting region				
Addis Ababa	808	47(5.8)	690(85.4)	71(8.8)
Amhara	1,989	948(47.7)	958(48.2)	83(4.2)
Benishangul-Gumuz	72	32(44.4)	37(51.4)	3(4.2)
Dire Dawa	38	8(21.1)	25(65.8)	5(13.2)
Gambella	4	0(0.0)	4(100.0)	0(0.0)
Harari	21	0(0.0)	19(90.5)	2(9.5)
Oromia	568	321(56.5)	232(40.9)	15(2.6)
Sidama	96	40(26.0)	104(67.5)	10(6.5)
SNNPR	154	12(12.5)	73(76.0)	11(11.5)
Somali	64	9(14.1)	48(75.0)	7(10.9)
Year of reporting				
2018	448	102(22.8)	313(69.9)	33(7.4)
2019	782	330(42.2)	440(56.3)	12(1.5)
2020	879	374(42.6)	456(51.9)	49(5.6)
2021	1,705	611(35.8)	981(57.5)	113(6.6)

### Sociodemographic characteristics of the mothers of the deceased perinates

The average age of the mother whose newborn was deceased during the early neonatal period was 27.3(with SD of 5.1) years. Similarly, the average maternal parity among mothers of the deceased perinate during the early neonatal period was 2.3 (with SD of 1.6). The proportion of early neonatal death was higher among women who attended secondary education and above (68.9%) as compared to women who had no education (48.8%). Likewise, mothers with no identified condition had a high proportion of early neonatal death (63.5%) as compared to mothers who had a complication of pregnancy during delivery (31.0%) (Table 5).

**Table 5:** Selected background characteristics of the deceased perinate’s mother by the timing of death in Ethiopia,2021.

Characteristic	Overall, N = 3,814	Occurrence of perinatal death		
		Stillbirth (n = 1417)(%)	Early neonate death (n = 2190)(%)	Late neonate death(n = 207)(%)
Maternal age				
Mean (SD)	3,814	27.4(5.7)	27.3(5.1)	27.0(5.4)
<35	3,309	1188(35.9)	1937(58.5)	184(5.6)
≥35	505	229(45.4)	253(50.1)	23(4.6)
Maternal parity				
Mean (SD)	3814	2.6(1.9)	2.3(1.6)	2.2(1.5)
<5	3,401	1208(35.5)	2001(58.8)	192(5.7)
≥5	413	209(50.6)	189(45.8)	15(3.6)
History of ANC follow up				
No	641	280(43.7)	326(50.9)	35(5.5)
Yes	3,173	1137(35.8)	1864(58.8)	172(5.4)
Maternal educational status				
Illiterate	2,168	1015(46.8)	1058(48.8)	95(4.4)
Primary	941	221(23.5)	646(68.7)	74(7.9)
Secondary and above	705	181(25.7)	486(68.9)	38(5.4)
Religion				
Christian	2,978	1149(38.6)	1675(56.3)	154(5.2)
Muslim	812	262(32.3)	500(61.6)	50(6.2)
Traditional	24	6(25.0)	15(62.5)	3(12.5)
Maternal health condition				
Other complications of labour and delivery	140	73(52.1)	64(45.7)	3(2.1)
Maternal medical and surgical conditions	295	96(32.5)	187(63.4)	12(4.1)
Maternal complications of pregnancy	326	217(66.6)	101(31.0)	8(2.5)
Complications of placenta, cord, and membranes	389	226(58.1)	146(37.5)	17(4.4)
No maternal conditions identified	2,664	805(30.2)	1692(63.5)	167(6.3)
Status of the mother				
Died	461	305(66.2)	145(31.5)	11(2.4)
Alive	3,353	1112(33.2)	2045(61.0)	196(5.9)

### Selected characteristics of the deceased perinate

The average gestational week of the deceased newborn during the early neonatal period was 35.3 (with SD of 3.6). The proportion of early neonatal death was higher among perinates who were delivered by cesarean section (73.1%) as compared to perinates delivered by spontaneous vaginal delivery (54.7%). Besides, perinates who were born in health facilities had a high proportion of early neonatal death (59.3%) as compared to perinates who were born in transit (25.4%). In addition, the proportion of early neonatal death was higher among newborns who died at a health facility (62.6%) as compared to perinates who died at home (18.9%) (Table 6).

**Table 6:** Selected characteristics of the deceased perinate by the timing of death in Ethiopia,2021.

Characteristic	Overall, N = 3,814	Occurrence of perinatal death		
		Stillbirth (n = 1417)(%)	Early neonatal death (n = 2190)(%)	Late neonatal death(n = 207)(%)
Estimated gestational age				
Mean (SD)	3,814	36.0(3.0)	35.3(3.6)	35.3(3.5)
Preterm(<37weeks)	2,318	837(36.1)	1362(58.8)	119(5.1)
Term (≥37 weeks)	1,496	580(38.8)	828(55.4)	88(5.9)
Sex of the perinate				
Female	1,614	635(39.3)	888(55.0)	91(5.6)
Male	2,200	782(35.6)	1302(59.2)	116(5.3)
Mode of delivery				
Caesarean section	520	106(20.4)	380(73.1)	34(6.5)
Operative vaginal delivery	223	84(37.7)	130(58.3)	9(4.0)
Spontaneous vaginal delivery	3,071	1227(40.0)	1680(54.7)	164(5.3)
Place of birth				
Health facility	3,478	1220(35.1)	2063(59.3)	195(5.6)
Home	265	146(55.1)	109(41.1)	10(3.8)
On transit	71	51(71.8)	18(25.4)	2(2.8)
Place of death				
Health facility	3,348	1055(31.5)	2096(62.6)	197(5.9)
Home	354	279(78.8)	67(18.9)	8(2.3)
On Transit	112	83(74.1)	27(24.1)	2(1.8)
Specific time of perinatal death				
Antepartum stillbirth	203	203 (100.0)	0 (0.0)	0 (0.0)
Still birth of unknown time	548	548 (100.0)	0 (0.0)	0 (0.0)
Intrapartum stillbirth	666	666 (100.0)	0 (0.0)	0 (0.0)
Neonatal	2,397	0(0.0)	2190(91.3)	207(8.7)

### Assigned cause of death to the deceased perinate

The proportion of early neonatal death was higher among newborns who died due to convulsions and disorders of cerebral status, where all the deaths were in the early neonatal period (100.0%), followed by complications of intrapartum events (96.0%), and low birth weight and prematurity (92.4%) (Table 7).

**Table 7:** Assigned cause of death to the deceased perinate by the timing of death in Ethiopia,2021.

Cause of death	Overall, N = 3,814	Occurrence of perinatal death		
		Stillbirth (n = 1417)(%)	Early neonatal death (n = 2190)(%)	Late neonatal death (n = 207)(%)
Antepartum death of unspecified cause	1	1 (100.0)	0 (0.0)	0 (0.0)
Other specified antepartum disorders	2	2 (100.0)	0 (0.0)	0 (0.0)
Intrapartum death of unspecified cause	2	2 (100.0)	0 (0.0)	0 (0.0)
Convulsions and disorders of cerebral status	4	0 (0.0)	4 (100.0)	0 (0.0)
Birth trauma	18	8(44.4)	5(27.8)	5(27.8)
Acute antepartum events	25	25 (100.0)	0 (0.0)	0 (0.0)
Other neonatal conditions	25	0 (0.0)	22 (88.0)	3 (12.0)
Respiratory and cardiovascular disorders	110	0 (0.0)	94(85.5)	16(14.5)
Congenital malformations, deformations, and chromosomal abnormalities	267	139 (52.1)	116 (43.4)	12(4.5)
Disorders related to fetal growth	274	274 (100.0)	0 (0.0)	0 (0.0)
Acute intrapartum events	339	339 (100.0)	0 (0.0)	0 (0.0)
Complications of intrapartum events	682	0 (0.0)	655 (96.0)	27 (4.0)
Low birth weight and prematurity	946	0 (0.0)	874 (92.4)	72 (7.6)
Infection	1,119	627 (56.1)	420 (37.5)	72(6.4)

### Delay factor for perinatal death

The proportion of early neonatal death due to delay one was higher among perinates whose mothers delayed seeking care due to previous bad experiences in health facilities (72.7%) than perinates whose mothers were delayed in seeking care due to lack of decision to a health Facility (42.5%). Similarly, the proportion of early neonatal death because of delay two was higher among perinates whose mothers were delayed reaching care due to the absence of a health facility with reasonable distance (54.0%) as compared to perinates whose mothers were delayed to reach care due to expensive cost of transportation (35.8%). Moreover, the proportion of early neonatal death because of delay three was higher among newborns who were not assessed and treated properly (74.7%) than newborns who travelled for a longer duration to get into the referred facility (64.0%) (Table 8).

**Table 8:** Delay factors contribute to perinatal death by the timing of death in Ethiopia,2021.

Delay factors	Occurrence of perinatal death			
	Overall, N = 3,814	Stillbirth (n = 1417)(%)	Early neonatal death (n = 2190)(%)	Late neonatal death(n = 207)(%)
Delay 1 –Decision to seek care				
Family poverty	257	74(28.8)	157(61.1)	26(10.1)
Bad experience with previous health care	11	2(18.2)	8(72.7)	1(9.1)
Failed to recognize the danger signs of pregnancy	1,104	501(45.4)	545(49.4)	58(5.3)
Unaware where to go	54	26(48.2)	25(46.3)	3(5.6)
Had no one take care of other children	50	20(40.0)	23(46.0)	7(14.0)
Reliant on traditional practice	37	14(37.8)	20(54.1)	3(8.1)
Lack of decision to health facility	454	246(54.2)	193(42.5)	15(3.3)
Delay 2 –Reaching care				
Absence of transportation	227	97(42.7)	121(53.3)	9(4.0)
Expensive cost of transportation	53	33(62.3)	19(35.9)	1(1.9)
No facility within reasonable distance	126	53(42.1)	68(54.0)	5(3.9)
Poor road condition	103	52(50.5)	47(45.6)	4(3.9)
Delay 3 –Receiving care				
Long travel time from health facility to health facility	760	224(29.5)	486(64.0)	50(6.6)
Long waiting time before treatment was received	319	91(28.5)	213(66.8)	15(4.7)
Mistakes during the assessment, diagnosis, and treatment	87	13(14.9)	65(74.7)	9(10.4)
Shortage of equipment and supplies	220	42(19.1)	158(71.8)	20(9.1)

### Bayesian multilevel parametric survival model

Bayesian multilevel parametric survival model was used to determine the factors that affect early neonatal death. In the analysis, both individual and facility-level factors were associated with early neonatal death.

#### Predictor of early neonatal death

Gestational age, assigned cause of death, delay one, type of health facility, residence, and delay three, were significant predictor variables at 95% Bayesian credible intervals (CrI).

As the perinate’s gestational age increase by one week the risk of death during the early neonatal period is reduced by 6% [ATR = 0.94,95%CrI:(0.93–0.96)]. Furthermore, as the score of delay one increases by one unit the risk of death during the early neonatal period increases by 4% [ATR = 1.04,95%CrI:(1.01–1.07)].

The risk of death due to birth injury during the early neonatal period was 2 times higher [ATR = 2.05,95%CrI:(1.30–3.32)] than the risk of death due to infection. In line with this, perinates who died due to complications of the intrapartum period had a 13% [ATR = 0.87,95%CrI:(0.83–0.90)] lower risk of death during the early neonatal period as compared to perinate who died due to infection.

As compared to primary health care, being treated in secondary and tertiary health care providers increases the risk of death during the early neonatal period by 14% [ATR = 1.14,95%CrI:(1.02–1.27)] and 15% [ATR = 1.15,95%CrI:(1.04–1.25)] respectively. Perinates whose mothers reside in a rural area had 3 [ATR = 3.53,95%CrI:(3.34–3.69)] times more risk of death during the early neonatal period than perinates whose mothers’ dwell in an urban area. Moreover, as the score of delay three increases by one unit, the risk of death during the early neonatal period increases by 21% [ATR = 1.21,95%CrI:(1.15–1.27)] (Table 9).

**Table 9:** Bivariable and multivariable Bayesian multilevel parametric survival model on predictors of days of early neonatal death in Ethiopia,2021.

Characteristics	Null model 1^a^	Model 2^b^	Model 3^c^	Model 4^d^
		Individual characteristics	Health facility characteristics	Individual and facility level characteristics
		Posterior CTR (95% CrI)	Posterior CTR (95% CrI)	Posterior ATR (95% CrI)
Estimated gestational week		0.95(0.94,0.96)	_	0.94(0.93,0.96)
Assigned cause of death				
Infection ®		1		1
Convulsions and disorders of cerebral status		0.34(0.19,0.57)	_	0.56(0.30,0.99)
Birth trauma		1.99(1.23,3.00)	_	2.05(1.30,3.32)
Other neonatal conditions		1.13(0.81,1.53)	_	1.05(0.87,1.27)
Respiratory and cardiovascular disorders		0.96(0.83,1.11)	_	0.94(0.83,1.05)
Congenital malformations, deformations and chromosomal abnormalities		0.84(0.74,0.96)	_	0.92(0.84,1.02)
Complications of intrapartum events		0.80(0.74,0.85)	_	0.87(0.83,0.90)
Low birth weight and prematurity		0.92(0.85,0.98)	_	0.95(0.91,1.00)
Number of ANC visit		0.94(0.93,0.96)	_	0.99(0.98,1.01)
Maternal age		1.09(1.04,1.14)	_	1.02(0.98,1.06)
Score of delay one		1.14(1.07,1.21)	_	1.04(1.01,1.07)
Maternal health condition				
No maternal condition®		1		1
Other complications of labour and delivery		1.31(1.11,1.56)	_	0.94(0.79,1.10)
Maternal medical and surgical conditions		1.01(0.92,1.11)	_	0.97(0.89,1.05)
Maternal complications of pregnancy		0.93(0.83,1.04)	_	1.10(0.99,1.21)
Complications of placenta, cord, and membranes		1.20(1.10,1.32)	_	1.08(0.98,1.18)
Residence				
Urban ®			1	1
Rural		_	3.59(3.28,3.81)	3.53(3.34,3.69)
Type of health facility				
Primary ®			1	1
Tertiary		_	1.16(1.03,1.31)	1.14(1.02,1.27)
Secondary		_	1.11(0.98,1.26)	1.15(1.04,1.25)
Score of delay three		_	1.08(1.02,3.81)	1.21(1.15,1.27)

CTR (Crude time ratio), ATR (Adjusted time ratio)

^®^ Reference for the category of an independent variable

#### Random effect analysis

The health facility variance estimate of the random effects parameters in the Null model suggests that there is a large variation in days of early neonatal death between clusters. The inclusion of all covariates (Model 3) reduces the between-cluster variance substantially. Moreover, the reduction of Bayesian DIC from the Null model to Model 3 suggests that the full model best fits the data (Table 10).

**Table 10:** Cluster level random effects parameter estimates.

Random effect	Null model ^a^	Model_2^b^	Model _3^c^	Model_ 4^d^
Health facility variance (95Crl%)	0.19(0.12–0.27)	0.17(0.11–0.25)	0.03(0.01–0.05)	0.02(0.01–0.04)
Model fit statics				
Log-likelihood	-5374.30	-5244.47	-4550.53	-4380.10
DIC	10134.33	9941.10	8974.61	8638.43

## Discussion

This assessment is the first comprehensive analysis of PDSR data from 2018 to 2021 that focuses specifically on the timing of early neonatal death among reviewed perinatal deaths. Our finding revealed that the timing of early neonatal death was significantly determined by both individual (gestational age, cause of perinate’s death, and delay one) and facility (residence, type of facility, and delay three) level factors.

Per the study findings, more than half of the early neonatal deaths occur during the first two days after birth. This finding was parallel with studies conducted in Ethiopia [60] and Nepal [61]. This implies that the measures taken in the first two days after birth could substantially reduce early neonatal death.

The statistical analysis output revealed that gestational age is one of the important factors that determine early neonatal death. As the gestational age increases by one week, the newborn is more likely to survive during the early neonatal period. This finding corresponds well with studies conducted in Ethiopia (Gondar, Addis Ababa, Jimma, Durame, and Mekelle) [62–67], Ghana [68], China [27], and Brazil [69]. The possible reason might be the fact that gestational age is strongly related to fetal maturity. As the gestational age increases the fetus’s immune system will mature, which will pave the way to reducing complications due to prematurity. On other hand, the pooled prevalence of preterm birth in Ethiopia was 12% with significant variation among regions [70]. Pregnancy-induced hypertension, short inter-pregnancies interval, and anemia were the common maternal risk factor for preterm birth in Ethiopia [33, 71, 72], these risks can be managed by improving access and utilization of contraception along with adequate ANC service provision. The supply of antenatal corticosteroids and thermal care together with robust resuscitation capacity are some of the recommendations for a better outcome for neonates since nearly half of preterm infant death in Ethiopia is attributed to respiratory distress syndrome [73].

Birth injury, infection, and complications of intrapartum events had a vital role in the survival of the early neonatal period. Birth injury is structural damage or a functional deterioration due to mechanical injury during labor and delivery [74]. The study revealed that the risk of death due to birth injury was much higher compared to infection. The finding was coherent with studies conducted in India [75] and Nigeria [76]. This might be partly related to cranial injuries of the neonate, which result in hemorrhage, increasing the risk of encountering high intracranial pressure, coagulopathy, and hypovolemic shock [77]. The combination of all the aforementioned conditions deteriorates the health status of the neonate resulting in death. On the other hand, birth injury is determined by three factors; namely fetal (fetal presentation and prematurity), maternal (poor uncontrolled maternal diabetics) and delivery mechanism (forceps and vacuum delivery) [78]. Therefore, the magnitude of birth injury can be used as a proxy indicator to monitor the quality of health service provision in the country [79].

A complication of intrapartum events, which is commonly defined as birth asphyxia, is one of the commonest causes of neonatal death within the first week of birth. The finding was parallel with studies conducted in Eritrea [80], Kenya [81], Tanzania, [82], Ghana [83], and Brazil [84]. This might be explained by fetal hypoxia, which affects the exchange and transport of oxygen from mother to the fetus during delivery; compromising the oxygen supply to the vital organ of the fetus, resulting in irreversible organ damage and death [85, 86]. In Ethiopia, birth asphyxia is one of the leading causes of neonatal morbidity and mortality [87]. Birth asphyxia-induced death demonstrates the presence of a noticeable gap in ANC follow-up, fetal monitoring during labor, and initiation of resuscitation of the neonate after delivery [88]. Thus, the finding clearly shows that managing potential maternal (diabetics, pregnancy-induced hypertension, anemia, and infection) factors during ANC visits should be coupled with improving facility readiness on fetal monitoring and neonatal resuscitation, as this could improve neonate’s chance of survival during the early neonatal period [89].

The study also revealed that neonatal infection has a vital role in the survival of neonates. The finding was coherent with studies conducted in Ethiopia (Dessie, Gondar, Jimma, Gama Gofa, and Tigray) [28, 65, 90–93] and Uganda [94]. This might be explained by neonatal sepsis resulting in septic shock and multiple organ dysfunction, eventually leading to death [95]. Neonatal sepsis causes nearly three-fourth of the morbidity and mortality during the early neonatal period in Ethiopia [96], and the causative agent for neonatal sepsis differs based on the onset of the sepsis. Early-onset sepsis (onset within 72 hours of birth) is thought to be caused by pathogens vertically transmitted from mothers while late-onset sepsis (beyond 72 hours) is usually attributed to pathogens acquired horizontally from the environment and/or caregivers [97]. Klebsiella, Staphylococcus aureus, Coagulase-negative Staphylococcus, and Escherichia coli are the leading cause of pathogens of neonatal sepsis in developing countries [98]. Overall, the finding implied that coordinated effort is needed in improving maternal health from pre-conception and ANC visits to post-delivery, along with the provision of clean and hygienic delivery services [99].

Our study also revealed that delay one has a major role in neonatal death, as the score of delay one increases by one unit, the neonate will have less chance of survival during the early neonatal period. A similar finding was observed in studies conducted in Rwanda [100], Zimbabwe [23], and Brazil [37]. The possible explanation is related to the lack of awareness about the danger signs of neonates’ illness and a preference for traditional remedies. This obviously will result in complicating mild and easily treatable illnesses to more severe cases and unwanted outcomes. On the contrary, Ethiopia has introduced and enacted a health extension program, which is designed to provide health education and basic health services at a community level [8]. The basic services delivered through the package of integrated community case management (ICCM) and community-based newborn care (CBNC) allow health extension workers (HEWs) to manage the major causes of child and newborn death at a grass-root level. However, the finding indicated that there is still a notable gap in the implementation of the programs. Therefore, the HEW program should be revitalized, and if possible integrated with other programs aimed at improving community awareness, through new communication technologies that address the first delay and improve neonatal outcomes.

The residence of the mother has a significant role in the survival of the neonate during the early neonatal period. Neonates born from mothers who live in rural areas are less likely to survive during the early neonatal period compared to their counterparts residing in urban areas. This finding was aligned with studies conducted in Ethiopia [30], Burundi [101], and Nigeria [31]. This might be due to the women, who live in rural areas, inability to acquire health care services comprehensively, and the lower chance of being informed about the danger sign and complications of pregnancy, labor, and delivery. Furthermore, cultural behaviors in rural areas have a great impact on the nutritional status of the women, as it could result in the prohibition of essential foods and drinks. According to the Ethiopian Service Availability and Readiness Assessment (SARA) report, rural health facilities have better service availability in Basic Emergency and Essential Obstetric and Newborn care (BEm/EONC) than urban health facilities; however, rural facilities have a low level of readiness to provide the services [102]. Overall, the finding implies that a concerted effort is needed in narrowing down the disparity between rural and urban areas by providing trained manpower, equipment, medicines, and commodities along with measures that will improve the health-seeking behavior of the rural community.

The study has also highlighted that the type of health facility and delay three had a significant role in the survival of newborns during the early neonatal period. Newborns treated in secondary and tertiary health facilities were less likely to survive than newborns treated in a primary health facility. Moreover, as the score of delay three increases by one unit, the newborn will be less likely to survive during the early neonatal period. This finding corresponded well with studies carried out in Ethiopia [103], Rwanda [104] Tanzania [36], and, Brazil [37]. This might be explained by the fact that poor follow-up of the newborn’s status after birth along with late initiation of neonatal intervention will result in irrevocable organ damage and death. Moreover, secondary, and tertiary health facilities are deemed to have better manpower and service than primary health facilities, as evidenced by the Ethiopian SARA report which indicated that, secondary and tertiary level health facilities have better availability and readiness in the provision of BEm/EONC services [102]. Thus, the high probability of death during the early neonatal period in secondary and tertiary level facilities could be explained by the presence of poor referral mechanisms, which are commonly defined by their limitations on transport, referral communication, and adherence to referral protocols [105], and the limited managing capacity at the lower-level health facilities. Overall, this finding suggests that the country has a long way to go in improving health facility’s readiness in managing neonatal complications at a lower level along with enhancing the referral linkage between facilities.

The study has limitations that needs to be acknowledged and the study findings should be interpreted meticulously in consideration of the gap listed below.1) Most of the reviewed perinatal death included in this study were facility death with limited inclusion of community death, which might introduce potential bias to the study. 2) Relatively small number of the expected facilities were actively involved in the full implementation of the system, which might introduce potential bias by obscuring the true burden. 3) Nearly all deaths were reported and reviewed from public facilities with limited involvement of private health facilities, and this could also affect the representativeness of the study.4) A small number of perinatal deaths were captured by the system, which is against national estimates and might compromise the inclusiveness of the study.

## Conclusion

Almost six in ten early neonatal deaths occur during the first two days after birth. Gestational age, cause of death (birth injury, infection, and birth asphyxia), and delay one were individual-level factors that determine the survivability of neonates during the early neonatal period; while residence, type of health facility, and delay three were identified as facility-level factors. Targeted intervention should be provided on the three leading causes of early neonatal death (birth injury, infection, and birth asphyxia). Revitalizing health extension programs should be taken as the forefront task to improve the health-seeking behaviour of mothers in combination with other parallel health promotions programs such as media campaigns and community mobilization. Moreover, strategies should be designed to improve maternal health conditions before delivery through pre-conception care and ANC visit to avoid the complication of preterm delivery. This should also be coupled with an intervention focused on enhancing the health-seeking behavior of mothers and narrowing down inequities in service access and provision between rural and urban residents. In line with this, facility readiness and referral linkage should be a top priority to reduce the burden of early neonatal death.
